# A Bull Gore Penetrating Injury to the Neck Presenting With Esophageal Perforation: A Case Report

**DOI:** 10.7759/cureus.23563

**Published:** 2022-03-28

**Authors:** Venkata Vineeth Vaddavalli, Charan Singh, Kishore Abuji, Lileswar Kaman, Ajay Savlania

**Affiliations:** 1 Department of General Surgery, Postgraduate Institute of Medical Education and Research, Chandigarh, IND

**Keywords:** cervical esophageal injury, emergency surgery, penetrating trauma neck, trauma, bull gore injury

## Abstract

Bull gore injuries are not uncommon in regions where bulls are commonly used for agriculture or as part of sporting culture. Most bull gore injuries occur in the trunk and extremities. Cervical bull gore injury might be due to penetrating or blunt trauma. In the neck, it may injure vital structures such as the trachea, esophagus, and carotid artery. Traumatic cervical esophageal injury is a rare entity. Prompt diagnosis and early intervention are essential for better outcomes. Delayed diagnosis (>24 hours) is associated with a mortality of 40-66%. Here, we report a case of a bull gore injury to the neck where the patient presented to our trauma bay after seven days. On evaluation, he was diagnosed with cervical esophageal injury and treated with primary repair of the esophagus over a T-tube and a feeding jejunostomy. He recovered well and was doing well on follow-up.

## Introduction

Bull gore injuries are not uncommon in rural areas of tropical countries where cattle are used for agriculture. Most bull gore injuries occur to the trunk and extremity, with head and neck injuries varying from 3.1% to 19% [1]. They can result in either penetrating or blunt trauma. Penetrating injury to the neck might result in esophageal perforation [2]. Traumatic esophageal injuries are rare, and delay in diagnosis can lead to increased morbidity and mortality. Here, we report a case of bull gore injury to the neck with cervical esophageal perforation with delayed presentation. The patient was operated on with primary repair over a T-tube and a feeding jejunostomy and recovered well.

## Case presentation

**Figure 1:**
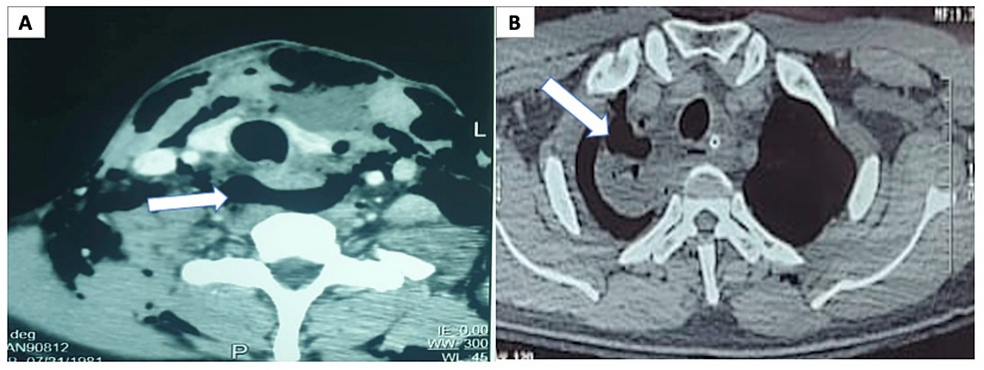
(A) Cross-sectional imaging showing pneumomediastinum (white arrow). (B) Cross-sectional imaging showing paraesophageal collection with air foci (white arrow).

**Figure 2:**
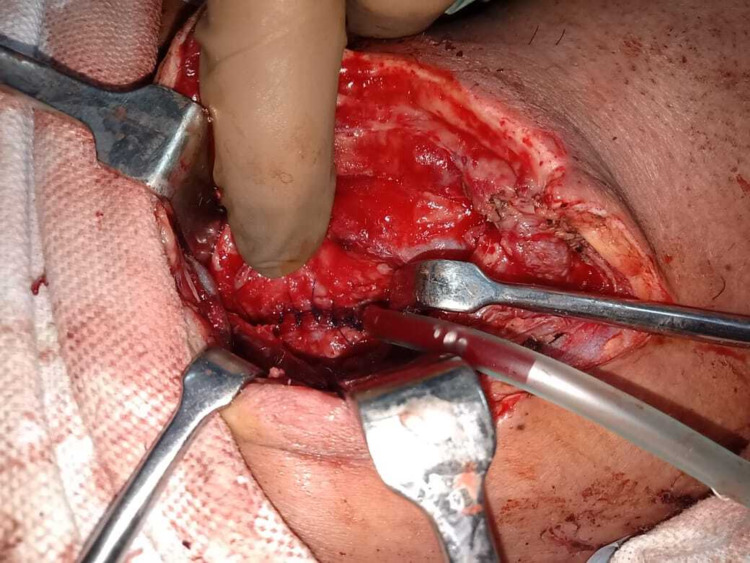
Intraoperative image showing primary repair of the esophageal perforation over T-tube.

A 39-year-old gentleman, a farmer by occupation, sustained a bull gore injury to the neck. He presented seven days later to our hospital with a complaint of swelling in the neck and purulent discharge from the wound. On the primary survey, his airway, breathing, and circulation were normal at presentation. On examination, erythema around the neck wound was present, and crepitus was noted on palpation. His vitals were stable, and cross-sectional imaging (CT scan) without oral contrast of the neck and chest revealed pneumomediastinum and paraesophageal collection with air foci (Figures 1A, 1B). Esophageal perforation was diagnosed, and surgical exploration was done after adequate resuscitation. Intraoperatively, there was a perforation in the anterior and posterior walls of the cervical esophagus. Trachea and neurovascular structures were normal, and 100 mL of purulent fluid was drained. Primary repair of the esophageal perforation using interrupted polydioxanone 4-0 sutures was done over a T-tube (Figure 2). A closed suction drain was placed in the anterior mediastinum, and a feeding jejunostomy (FJ) was performed.

**Figure 3:**
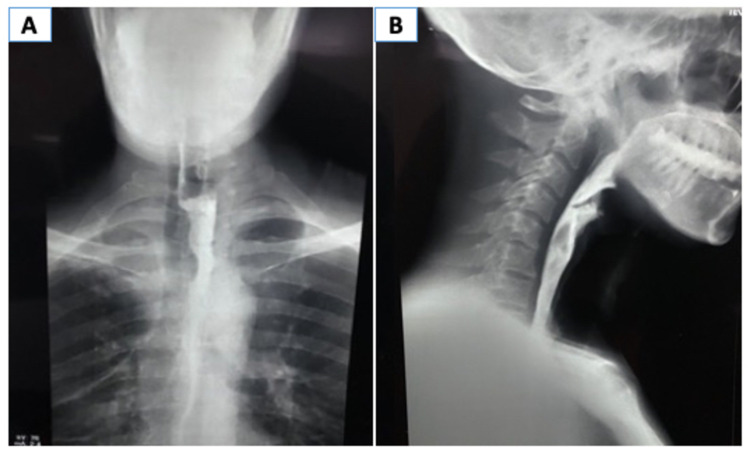
Oral gastrografin study on follow-up. (A) Anteroposterior view; (B) lateral view.

Postoperatively, the patient was kept nil per oral and enteral feed was given through FJ. On postoperative day three, he developed febrile spikes, and a high-resolution CT scan of the chest showed a paraesophageal collection, which was drained percutaneously. The mediastinal drain was removed when the output was minimal, and the T-tube was removed on the 10th postoperative day. After two weeks, an oral gastrografin study was performed, which showed no contrast extravasation or any narrowing of the esophagus (Figure 3). The patient was then allowed oral feeding, and he tolerated it well. The patient was doing well at the 18-month follow-up.

## Discussion

Bull gore injuries to the neck can result in penetrating or blunt trauma. The contamination of bull gore results in a higher incidence of wound complications [2]. Patients can present with neck swelling, dysphagia, or respiratory distress. There can be underlying life-threatening complications such as internal carotid artery injury, severe tracheobronchial injury, and air embolism [1]. In low-resource settings and lack of emergency care, the management of bull gore injuries can be challenging. Hence, prompt referral to a tertiary care center is necessary. In this case, the patient presented late to our center, and cervical esophageal perforation was diagnosed on evaluation.

Esophageal injuries are rare, and traumatic esophageal perforation constitutes 15% of esophageal injuries [3]. Delayed diagnosis (>24 hours) is associated with a 40-66% mortality [4]. There is an increased risk of mediastinitis, abscess, or empyema formation from the leakage of its contents because of a delay in diagnosis [5]. Hence, early diagnosis and prompt surgical treatment are necessary for improved outcomes. Diagnosis of esophageal perforation is based on clinical suspicion and imaging techniques. A plain X-ray of the neck may show subcutaneous emphysema, pleural effusion, lung condensation, pneumomediastinum, or pneumothorax [6]. The most sensitive method for diagnosing esophageal injury is a CT scan. Typical findings on a CT scan include paraesophageal collection, extraluminal air, esophageal thickening, esophageal fistula, and communication of an adjacent mediastinal air-fluid collection with the air-filled esophagus [7]. Fluid resuscitation and broad-spectrum antibiotic coverage should be started immediately when a diagnosis is made. Primary repair is the procedure of choice for hemodynamically stable patients who present early. In addition, reinforcement with a muscle flap can be done. Drainage alone or T-tube placement can be done in patients who present late because of severe contamination and inflammation. Non-operative management can be done in patients with no signs of sepsis and contained leak [8]. In our case, the patient presented late, and we performed a primary repair over T-tube with an FJ because the esophageal wall was edematous and friable.

## Conclusions

Bull gore injuries are not uncommon in areas where bulls are used for agriculture or sporting purposes. Sometimes, they can be life-threatening, leading to increased morbidity among patients. Bull gore injuries leading to penetrating or blunt trauma of the neck are infrequent. A high index of suspicion is needed to rule out cervical esophageal, tracheal, and vascular injuries in case of neck trauma by a thorough evaluation. Delayed diagnosis leads to increased morbidity and mortality. Our patient presented late with cervical esophageal injury. In such scenarios, as the esophageal wall will be friable, primary repair over T-tube with an FJ for enteral nutrition is a good option for optimal clinical outcomes.
